# New perspectives in pediatric dialysis technologies: the case for neonates and infants with acute kidney injury

**DOI:** 10.1007/s00467-023-05933-x

**Published:** 2023-04-04

**Authors:** Mattia Parolin, Giovanni Ceschia, Enrico Vidal

**Affiliations:** 1 Pediatric Nephrology Unit, Department for Women’s and Children’s Health, University-Hospital of Padua, Padua, Italy; 2 Department of Medicine (DAME), University of Udine, Udine, Italy

**Keywords:** Neonates, Infants, AKI, Dialysis, CKRT

## Abstract

Advancements in pediatric dialysis generally rely on adaptation of technology originally developed for adults. However, in the last decade, particular attention has been paid to neonatal extracorporeal therapies for acute kidney care, an area in which technology has made giant strides in recent years. Peritoneal dialysis (PD) is the kidney replacement therapy (KRT) of choice in the youngest age group because of its simplicity and effectiveness. However, extracorporeal blood purification provides more rapid clearance of solutes and faster fluid removal. Hemodialysis (HD) and continuous KRT (CKRT) are thus the most used dialysis modalities for pediatric acute kidney injury (AKI) in developed countries. The utilization of extracorporeal dialysis for small children is associated with a series of clinical and technical challenges which have discouraged the use of CKRT in this population. The revolution in the management of AKI in newborns has started recently with the development of new CKRT machines for small infants. These new devices have a small extracorporeal volume that potentially prevents the use of blood to prime lines and dialyzer, allow a better volume control and the use of small-sized catheter without compromising the blood flow amount. Thanks to the development of new dedicated devices, we are currently dealing with a true “*scientific revolution*” in the management of neonates and infants who require an acute kidney support.

## Introduction

**Fig. 1:**
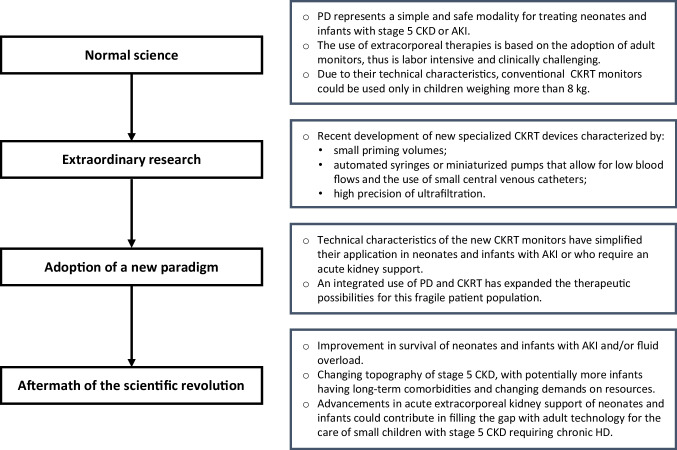
Sequential steps for the paradigm shift in the dialysis treatment of neonates and infants with AKI or who require an acute kidney support

In his book “*The Structure of Scientific Revolutions*” published in 1962, Thomas Kuhn, an American philosopher of science, introduced the concept of “paradigm shift” [1]. This occurs when a paradigm that dominates a scientific field becomes incompatible with new scientific discoveries triggering a process that ends with the adoption of a new theory or paradigm. The shift requires the progression toward four sequential steps that eventually lead to a scientific revolution: I. normal science; II. extraordinary research; III. adoption of a new paradigm; IV. aftermath of the scientific revolution (Fig. 1).

Kidney replacement therapies (KRT) in children are currently well-established procedures, both in the acute and chronic settings. In the late 1960s, PD started to be used for acute kidney injury (AKI) in pediatric patients, whereas the first chronic PD programs began in the early 1970s. The period from 1960 to 1970, the so-called first development decade, also saw preliminary experiences in the clinical use of chronic HD in children through pumpless systems. Since then, advances of KRT in children have occurred, although not at the same pace with adults. Data from European Society of Pediatric Nephrology (ESPN)/European Renal Association (ERA) Registry indicate that, from 2007 to 2016, incidence of children aged < 15 years commencing chronic KRT remained stable, ranging from 5.5 per million age-related population (pmarp) to 6.6 pmarp, whereas prevalence increased from 26.4 pmarp in 2007 to 32.1 pmarp in 2016 [2]. Given an estimated pediatric European population (0–15 years) of about 120 million, this corresponds to an overall number of 3200–3900 patients treated. According to the ERA Registry, as of December 31, 2019, prevalent patients on KRT > 19 years of age were approximately 600,000, a figure that is 150–200 times higher than that of children [3]. These numbers explain why, in the end, advancements in pediatric dialysis rely on adaptation of technology originally developed for adults. This applies for both PD and HD, in the acute and chronic setting. Despite an improvement over the years in safety and efficacy of dialysis procedures in children, still some problems persist when adult technology is adapted to children, and this is further emphasized when treating infants.

However, in the last decade, particular attention has been focused on neonatal dialysis, especially in acute extracorporeal therapies, where most of the innovations have been concentrated and a scientific revolution is likely occurring. In this review, we aim to analyze the four sequential steps that, in our opinion, have led to a paradigm shift in the dialysis treatment of neonates and infants with AKI or who require an acute kidney support because of fluid overload, electrolyte imbalances, or metabolic syndromes.

## The normal science

KRT in neonates and infants is one of the most challenging and demanding treatments in nephrology. PD is the method of choice for initiating KRT in the youngest age group in the context of both stage 5 chronic kidney disease (CKD 5) and AKI [4–8]. The popularity and success of this technique in neonatal age largely derive from its simplicity and effectiveness in even the smallest patients. PD has a low cost, avoids the need for vascular access and anticoagulation, provides excellent hemodynamic stability, and is often efficacious in terms of fluid and solute management. This dialysis modality has undergone technical updates over time, with development of new automated cyclers, allowing for a remote patient management and adjustment of therapy, such as in the case of adapted PD where an automatic sequence of dwells with different durations and fill volumes can be set to optimize both ultrafiltration (UF) and solute clearance [9]. However, the underlying PD technology remains quite simple with few engineering barriers to overcome (i.e., dialysis inaccuracy and poor drainage in case of fill volumes less than 100 ml, automatic and flexible mixing of low and high-dextrose concentration solutions to achieve adequate fluid removal). This makes PD easy to implement, requiring fewer technical skills and costs, and this modality is thus the most common home dialysis treatment [10]. Data from large international registries show that the vast majority of infants and small children with CKD 5 are currently treated with PD, whereas only 8–13.5% of patients younger than 1 year of age receive chronic HD [11–13].

However, extracorporeal blood purification therapies (HD and continuous kidney replacement therapy (CKRT)) provide faster fluid removal and, using both diffusion and convection, a more rapid clearance of small, middle, and larger molecular weight substances. These techniques are, therefore, more efficient for acute conditions, such as in the case of significant fluid overload, in hypercatabolic circumstances, inborn errors of metabolism, or when a toxin removal is required. Given the advantages on clearance and UF, the treatment of pediatric AKI in high-income countries is mainly based on the use of HD or CKRT instead of PD.

AKI is a common complication in intensive care units (ICUs), occurring in 20–30% of patients in pediatric ICU [14, 15] and in about 30% of critically ill neonates admitted to neonatal ICUs [16]. The definition of the neonatal modified Kidney Disease: Improving Global Outcomes (KDIGO) criteria has resulted in significant advances in the understanding of neonatal AKI in recent years [17]. Prematurity and low birth weight, perinatal asphyxia, necrotizing enterocolitis, patent ductus arteriosus, congenital heart disease, cardiac surgery, and the use of extracorporeal membrane oxygenation are all high-risk conditions for AKI and can lead to neonatal acute kidney dysfunction because of an ischemic injury or in the setting of medication-induced AKI [18].

Though the incidence of AKI is high among patients admitted in neonatal ICUs, the use of kidney support in neonates is still uncommon. According to data emerging from the AWAKEN study (Assessment of Worldwide Acute Kidney Injury Epidemiology in Neonates), 4% of infants with AKI (25 out of 605) received KRT, and only 0.7% were treated with CKRT or HD [16]. It should be underlined that, in the centers involved in the study, there was no specific KRT device for newborns and infants, an element that could have contributed to the low number of treatments.

The employment of extracorporeal dialysis in small children is associated with a series of clinical challenges, including difficulties of vascular access, hypotension on connection, hypothermia due to an immature temperature body control and thrombocytopenia [19]. These conditions discouraged the use of CKRT in this specific population, reserving its application mainly in case of a PD contraindication.

From a technical point of view, the following are probably the most important barriers to a safe CKRT in neonates and infants:

1. *The vascular access*: in very small children, well-functioning vascular access can be difficult to place; moreover, the rates of infectious and mechanical complications are very high [18]. Access diameter significantly affects circuit survival. In the prospective pediatric CKRT (ppCRRT) Registry experience, functional performances of catheters ranging from 7 to 9 French were similar, whereas none of the 5 French catheters used to provide CKRT lasted longer than 20 h [20]. However, complications of catheterization are more common in young infants due to technical challenges and because the catheter caliber used in these small patients is proportionately larger than in older children [21]. A higher ratio of catheter diameter to vessel size increases the risk of venous stenosis, which over time can affect dialysis options for neonates and infants who potentially have a long history of KRT ahead of them [22].
2. *The blood pump*: blood pumps integrated in conventional HD machines are characterized by two moving rollers normally placed at 180° interval that squeeze a flexible plastic tube, moving the fluid inside along in a specific direction. The resulting pressure necessitates adequate catheter blood flow that directly depends on catheter size dimensions. Thus, when conventional HD or CKRT machines are used with small-sized central venous catheters, dialysis kinetics might be inadequate, and a suboptimal rate of blood flow significantly increases the risk of clotting. Given the Poiseuille law, keeping constant the blood flow with a smaller size catheter requires a proportional increase in venous circuit pressures; when exceeding the safe threshold of + 200 mmHg, this might, in turn, result in several machine alarms that prevent treatment continuation.
3. *The extracorporeal volume*: the priming volume of the extracorporeal circuit should ideally not exceed 10% of a patient’s circulating blood volume, which is usually assumed to be 80–90 mL/kg in neonates and infants. The higher this ratio, the more the patient’s blood is decreased at the start of extracorporeal treatment, leading to an increased risk of cardiovascular instability. The initiation phase of extracorporeal treatment is widely recognized as a hemodynamically critical procedure. In a study of 174 children undergoing CKRT, low blood pressure at dialysis initiation was found in 53 patients (30.4%) [21]. When the priming volume exceeds 10% of the patient’s circulating volume, pre-filling the circuit with blood may reduce the risk of hemodilution and hemodynamic instability. Taking as an example a 5-kg infant requiring CKRT, his circulating blood volume would be approximately 400 mL. The pediatric sets available on current adult CKRT machines have a priming volume between 59 and 100 mL. Even the smallest circuit, therefore, has an extracorporeal volume greater than 40 ml, which is 10% of the patient’s blood volume. In this case, a blood prime would reduce the hemodynamic risk, however exposing the child to potassium and citrate overload, transfusion reactions, infectious risk, and sensitization due to HLA exposure.
4. *Fluid removal accuracy*: the relatively low body weight of patients and neonatal hemodynamics require a high precision in fluid balance assessment and monitoring during extracorporeal therapies. The better systemic fluid balance control in conventional HD and CKRT is set at ± 30 ml/h. Consequently, there are no adult monitors for continuous or intermittent kidney support currently approved for treating patients of low body weight (< 8 kg).

## Extraordinary research

The revolution in the management of neonatal AKI has recently started with the development of new CKRT machines adapted or specifically designed for young children. The new monitors have smaller circuits that reduce extracorporeal volume and limit the need for blood priming, have more precise control systems that minimize machine errors, provide smoother flow rate adjustment, and allow the use of smaller-sized catheters. In 1995, Everdell et al. reported the results of a manual syringe-driven HD technique to treat 3 babies weighing 630, 808, and 1140 g, using a single-lumen access line, and without the need for blood-priming [23]. This circuit is very simple and consists of two syringes, a small dialyzer (Minifltro, Amicon®), and a set of three-way stopcocks. The first syringe works as a reservoir, which allows blood to be slowly withdrawn from the patient but, at the same time, to make it flow quickly through the filter. The second syringe is held in suction by an elastic band, which generates a positive transmembrane pressure to achieve UF. This manual system was effective but extremely labor intensive and was then integrated in a first automated device, which would drive the system [24]. This device has been subsequently developed into the Newcastle Infant Dialysis and Ultrafiltration System (NIDUS®, Allmed, UK), designed to provide single-lumen HD and UF to children weighing between 800 and 8 kg [25]. This machine requires a 5-ml priming volume (plus stroke volume) and can be used with a 0.045-m^2^ filter, setting a dialysate flow rate in the range of 0–400 ml per hour and an UF rate in the range of 0–60 ml per hour. A recent study by Crosier et al. has shown that, when used in small infants, two modern CKRT devices with conventional circuits, the Prismaflex and Aquarius, do not have the capacity to deliver precise or reliable UF, or to record its volume correctly even when they are set to produce zero UF, but the NIDUS volumetrically controlled circuit does [26]. A clinical study is currently active in the UK to evaluate efficacy, safety, and outcomes of the NIDUS machine as compared with the other existing KRT methods for children who weigh < 8 kg.

In the first decade of the twenty-first century, Ronco and colleagues were working on wearable and miniaturized kidney replacement devices with the purpose of making them portable, thus prolonging the HD treatments as long as possible to improve the patient outcome. On the way to the wearable artificial kidney, new discoveries have been made, such as a complete system for HD in newborns [27]. The Cardio Renal Pediatric Dialysis Emergency Machine (CARPEDIEM™, Medtronic, MN) is designed to treat patients between 2.5 and 10 kg with filters of 3 different sizes (0.075, 0.17, and 0.29 m^2^), with a priming volume of 26, 32, and 41 ml, respectively. The CARPEDIEM monitor provides slow continuous ultrafiltration (SCUF), pre- or post-dilution continuous veno-venous hemofiltration (CVVH), continuous veno-venous hemodialysis (CVVHD), and plasma exchange, with an ultrafiltration error of 1 g/h [28]. The blood, the dialysis, or the replacement fluid and the effluent are pushed by a newly designed 3-roller miniaturized peristaltic pump with great accuracy, limiting hemolysis, and traumatic injury of the small circuit lines. The introduction of the miniaturized blood pump has significantly improved the survival of small central venous lines, avoiding pressure peaks at low fluxes but allowing comparable stroke volumes compared to adult pumps, thus minimizing the risk of circuit clotting phenomena [29, 30]. A series of experimental tests was performed, comparing a two-roller pump typical of standard adult machines and the small three-roller pump developed in the pediatric KRT device [30]. 4F and 5F catheters were tested, and the maximum blood flows obtainable were measured by maintaining the circuit pressures in the “safe” range, i.e., between − 100 and + 100 mmHg. Maximum flows of 13 and 29 mL/min, respectively, were achieved with the three-roller pediatric pump. On the contrary, the use of an adult roller pump with the same set of catheters allowed maximal flows up to 10 and 20 mL/min, respectively. Moreover, in-flow (out-flow) pressure profiles recorded close to the catheters showed fluctuations from a narrow range of − 50 to − 30 mmHg (+ 20–50 mmHg) for the small pump to a wider range of − 70 to − 25 mmHg (+ 5–65 mmHg) for the bigger device.

Aquadex™ (Nuwellis, MN) is a device developed for adult patients with congestive heart failure to control the fluid overload in a condition of diuretic resistance. This machine has been developed to perform only SCUF, without clearance, allowing simplification of the circuit, which, therefore, has a minimal priming volume (33 mL). In 2013, there were no devices specifically developed for small pediatric patients in the USA, and Askenazi and colleagues chose Aquadex™ as the smallest adult-based extracorporeal device to adapt it for neonates and infants. Therefore, a modified version was developed by adding a pre-filter replacement fluid, administered with an infusion pump through the access line, entirely removed as ultrafiltrate. The resulting dialysis mode was, therefore, a real CVVH, implementing the monitor with a convective clearance component [31]. Despite this device having the same accuracy as the other adult-based machines (fluid removal error of 10 g/h), the “modified” Aquadex has shown good performance and safety, becoming a new option for small children [32].

## Adoption of a new paradigm

**Fig. 2:**
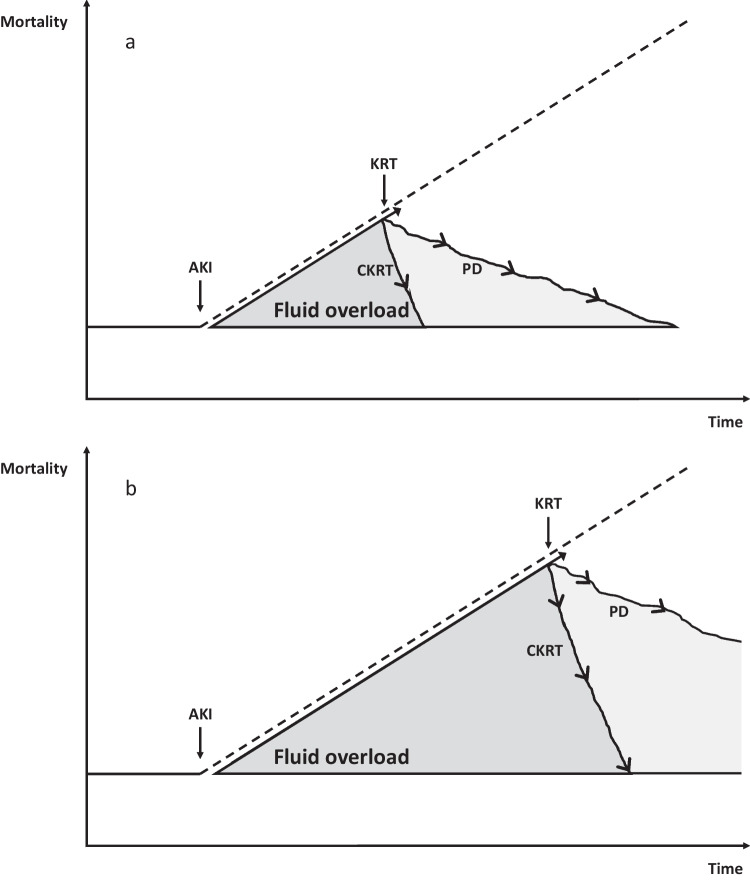
Early (**a**) or late (**b**) application of different kidney replacement therapy (KRT) modalities (PD vs. CKRT) impacts on patients’ survival

In recent years, there has been, for the first time in history, the conception, testing, and approval of dialysis devices specifically dedicated to small children. These new machines have been approved with varying time lines in most Western countries, bursting into the pediatric world, rapidly changing clinical practice. The new dialysis devices were initially used in patients in whom PD was contraindicated or proved ineffective, with adequate results on clearance, fluid removal, and safety. Clinicians quickly realized the versatility and precision of these extracorporeal treatments, which provided greater dialysis efficiency than PD and allowed for fluid management planning [25, 26, 33]. It is well-known that, in critically ill children with AKI, the amount of fluid overload has an almost linear association with mortality [34]. Children who undergo cardiac surgery with cardiopulmonary bypass are a group of patients at risk of AKI and fluid overload. In this setting, early initiation of PD has been associated with significantly better outcomes as compared to children treated when the fluid accumulation was already significant [35, 36]. This has prompted the use of detailed indications for preoperative planning and prophylactic placement of a PD catheter, with the aim of starting dialysis early based on clinical and laboratory predictors of AKI and before having a fluid overload that is refractory to conservative therapy [37]. In the setting of sepsis-associated AKI, early PD compared with standard PD also resulted in a favorable kidney outcome, decreased duration of PD, and early discontinuation of dialysis [38]. However, when the amount of fluid overload is already significant, the goal of an adequate KRT should be to remove the fluids as fast as possible to rapidly reduce the associated adverse outcome and mortality risk. The use of PD in hypercatabolic patients or those with splanchnic hypoperfusion or who are on vasopressors may potentially result in unpredictable fluid removal rates [39]. New specialized CKRT machines have precise volumetric systems that guide fluid removal, conferring clear advantages and likely contributing to a more efficient reduction of fluid overload as compared to PD (Fig. 2). An international survey about KRT modalities used in children > 12 years old with AKI from high-income countries, published in 2017, showed that preferred dialysis modalities were HD or CKRT (96%) instead of PD [40]. No specific data are available on neonates and small infants with AKI, which, however, would describe a situation prior to the development of new dedicated devices. The possibility of using extracorporeal machines with small central venous catheters, low priming volumes, small and precise increments in ultrafiltration, and weight loss will simplify their practical application. In centers where these devices are available, an integrated use of PD and CKRT has expanded the therapeutic possibilities and an international survey is soon needed to outline the new scenario. However, all the new devices are characterized by low dialysis and/or replacement fluids and are intended for CKRT. A gap in “current science” still exists when extracorporeal dialysis is needed as a maintenance and intermittent therapy in children with stage 5 CKD and a body weight < 10 kg. In this setting, there are currently no chronic dialysis monitors that are approved or could be applied.

## Aftermath of the scientific revolution

Despite differences in definition and staging, acute kidney dysfunction in critically ill patients is a risk factor for mortality, and AKI is a recognized “deadly syndrome.” In the AWAKEN study, infants with AKI had a mortality rate of 9.7%, significantly higher than those without AKI (1.4%) [16]. When AKI was classified according to its severity, infants with stage 3 had worse survival outcomes than infants with stage 2, stage 1, or no AKI. In neonates who underwent KRT, mortality rates of up to 60–80% are reported and are influenced by the intensive care setting, patient status, timing of dialysis start, amount of fluid overload, and dialysis modality [16, 41–43]. In a retrospective analysis comparing the CARPEDIEM™ registry and the ppCRRT registry, despite the low body weight and the high severity of illness, better survival rates to CKRT termination have been shown in newborns and small infants treated with a specifically dedicated device (97%) than those treated with adult-adapted machines (44%) [44].

The development and clinical application of these new tools would result in significant advancements in the care of the vulnerable and highly demanding patient population of neonates and infants with severe AKI or other conditions that could benefit from extracorporeal kidney support (i.e., sepsis with fluid overload, electrolyte abnormalities, acute liver failure or inborn errors of metabolism). As suggested by Tal et al., survival of more neonates with AKI could lead to a change in topography of the pediatric CKD 5 population, with more children having long-term comorbidities and changing demands on resources [45].

However, the use of this innovative technology is still precluded in resource-poor countries and, given the high complexity of the target patient population, should involve a multidisciplinary decision-making process. The possibility to integrate more KRT options for small children increases the demand for resources and will require adequate training and established protocols and procedures. A recent educational review provides detailed recommendations on how to build a comprehensive and integrated neonatal kidney support therapy program in those countries where technologies and resources are available [46].

There is also hope that the scientific revolution occurring in the field of neonatal and infant CKRT will open new perspectives in pediatric dialysis technology and raise awareness of companies about the importance of bridging the gap in chronic HD treatment of small children with CKD 5, currently still an “orphan disease.”

## Multiple choice questions

Answers are given following the reference list.

1. What is the safe threshold of priming volume for an extracorporeal dialysis treatment (as a percentage of the patient’s blood volume)?
  - 3%
  - 5%
  - 10%
  - 20%
2. What is one of the greatest technical innovations of the CARPEDIEM dialysis machine?
  - the three-roller pump, which allows for controlled flows even in small-sized catheters
  - the ability to perform CVVHD
  - the possibility of using the device with catheters of different sizes
  - the possibility to use three different filters
3. What makes a device, specifically designed for neonatal dialysis, safe and reliable?
  - the small size of the hardware
  - the accuracy of the scales and alarms
  - the ability to perform multiple types of extracorporeal treatments
  - the possibility of performing blood priming
4. What is one of the biggest challenges of neonatal CKRT when performed with adult dialysis machines?
  - the patient’s lack of compliance
  - the requested size of the vascular access and the need for circuit priming
  - the inadequate warming system for dialysis fluids
  - the absence of a dedicated fluid management monitoring tool
